# Trends and determinants of Emergency Room admissions for asthma: A retrospective evaluation in Northeast Italy

**DOI:** 10.1016/j.waojou.2019.100046

**Published:** 2019-07-03

**Authors:** Marco Caminati, Andrea Vianello, Giorgio Ricci, Giuliana Festi, Roberto Bellamoli, Sofia Longhi, Mariangiola Crivellaro, Guido Marcer, Marco Monai, Margherita Andretta, Chiara Bovo, Gianenrico Senna

**Affiliations:** a Asthma Center and Allergy Unit, Verona University and General Hospital, Verona, Italy; b Department of Medicine, University of Verona, Verona, Italy; c Respiratory Pathophysiology Division, University-City Hospital of Padua, Padua, Italy; d Emergency Department, Clinical Toxicology Unit, Verona University and General Hospital, Verona, Italy; e Respiratory Unit and Asthma Center, Verona University and General Hospital, Verona, Italy; f Allergy Service, Department of Medicine and Public Health, University of Padua, Padua, Italy; g Meteorological Service, Veneto Regional Agency for Environment Protection and Prevention, Padua, Italy; h Health Technology Assessment Unit, Azienda Zero, Padova, Italy; i Medical Direction, Verona University and General Hospital, Verona, Italy

**Keywords:** Asthma exacerbation, Emergency room admission, Asthma control, Asthma exacerbation determinants, ER, Emergency Room, ARPAV, Agenzia Regionale per la Prevenzione e Protezione Ambientale del Veneto, ISTAT, Italian National Institute of Statistics

## Abstract

**Background:**

Asthma still represents a cause of death and hospital admissions worldwide. Our study aimed at analyzing the trend of Emergency Room (ER) asthma admissions in Northeast Italy in order to investigate the relevance of specific patient-related determinants and environmental triggers (pollens, mold spores, and pollutants).

**Methods:**

Retrospective data from admissions for asthma exacerbations registered between the years 2013 and 2015 in two main ERs in Northeast Italy were collected. Data about patients' age, sex and nationality were recorded. Classification of disease severity followed the current Italian ER triage scoring system (white: no need for emergency treatment; green: need for fast treatment; yellow: severe condition; red: life-threatening condition). Data on pollen/mold spore counts and pollutants were analyzed.

**Results:**

Overall, 1745 ​ER admissions for asthma were registered, with a persistent and significant increase year by year. A slight prevalence of females and patients over 50 years old was observed. Immigrants accounted for 32%, 36% and 26% of admissions respectively in 2013, 2014 and 2015. The prevalence of immigrants' admissions was significantly higher when comparing the relative ratio of immigrant populations/Italian nationals (p ​< ​0.05). The admissions were coded as follows: white, 6.30%; green, 35.36%; yellow, 39.37%; red, 18.97%. People aged ≥50 years were more frequently admitted with a red code, but the trend was not statistically significant (p ​= ​0,0815). By contrast, amongst immigrants there was a higher prevalence of white and green codes observed in comparison with Italian nationals. Grass pollen peak and PM_10_ high levels represented environmental determinants of ER admissions increase.

**Conclusions:**

The increasing rate of asthma-related ER admissions highlights the need for implementing asthma control strategies. Investigating the traits of patients referring to ER for asthma exacerbations, as well as environmental-related determinants, may help in identifying at-risk individuals and in orienting preventive strategies accordingly. Immigrants represent the most vulnerable sub-population, and their potential difficulties in accessing treatments and health services should be specifically addressed. Overall, implementing patient education in order to improve treatment adherence, as well as providing an asthma action plan to every asthmatic patient, continue to be the most urgent needs.

## Background

Asthma control still represents an unmet need.^1^, ^2^ Asthma is a cause of death and hospital admissions worldwide.^3^ Conflicting data has been recently published concerning the association between asthma fatalities and asthma severity. Despite a decrease of registered asthma mortality in several countries,^4^, ^5^, ^6^, ^7^ a recent study evaluating trends of asthma mortality in 46 countries during a nine-year period (1993–2012) observed a general plateau in the last five years.^3^ Furthermore, according to some data coming from the U.S., the severity of the disease seems to decline; in fact, a progressive reduction of the hospitalizations over time has been described.^1^ However, the lack of asthma control still represents an unmet need both in severe^8^ as well as in mild asthma.^8^

Our group has recently reported a cluster of 16 cases of fatal asthma, registered from the years 2013 to 2015, that occurred in the Veneto region, which is the largest in Northeast Italy.^9^ The patients were young, and most of them were not on regular treatment but used short acting bronchodilators on demand only. However, in five cases a close relationship with high alternaria concentration was observed. The role of pollutants seemed to be less relevant.

Admissions to emergency room (ER), overuse of short acting beta 2 agonists and repeated oral steroid courses to treat exacerbations are the hallmarks of uncontrolled asthma. Among them, ER admissions represent the easiest and more reliable marker of asthma control at least within a specific geographical district; in fact, oral steroids or short acting bronchodilators may be prescribed in many diseases other than asthma.

The current study aims at analyzing the trend of ER asthma admissions in this region within a period of 3 years, in order to investigate the relevance of specific determinants in terms of specific clinical features and environmental triggers (pollens, mold spores, and pollutants, such as PM10, ozone and carbon monoxide).

## Methods

### Emergency Room Admissions

Retrospective data from admissions for asthma exacerbations registered from 1 January 2013 to 31 December, 2015 in ERs in Verona and Padua were collected. Verona and Padua represent the main towns of the Veneto region, where most of the cases of fatal asthma were registered.^9^ Demographic data recorded for each patient were: age, sex and nationality. Classification of the severity of disease was also collected as per the current Italian ER triage scoring system according to four different color codes (white: mild disease, no need for emergency treatment; green: not severe symptoms but in need of fast treatment; yellow: not life-threatening but severe condition; red: life-threatening condition).^10^ Data evaluating the population of the two towns and the prevalence of immigrants in both cities were obtained by ISTAT (National Institute of Statistics).^11^

### Pollen/mold spore counts

Daily pollen count is regularly performed by ARPAV (Agenzia Regionale per la Prevenzione e Protezione Ambientale del Veneto) for the whole region. Data for Verona and Padua were collected by two pollen traps for each town.^12^ The pollen count is recorded by a Hirst pollen trap (VPPS 2000; Lanzoni S.r.l., Bologna, Italy), which is specifically designed for sampling pollen and fungal spores. Flow rate is fixed and provided by an external vacuum pump. The airflow is of 10 ​L/min, and the speed of the trapping surface was 2 ​mm/h. Data are expressed as the average daily concentration from 0 to 24 ​h (n/m3). The pollen count values, relative to the scanned surface, were extrapolated to the entire surface of the sampling. Pollen monitoring is regularly carried out by ARPAV each year from 1st of February to 31st October. The monitored pollen species are: grass, Parietaria, Olea, Birch, Hazelnut, Cypressus, mugwort and ragweed. Among molds only Alternaria is examined, being the most relevant allergen in this territory. The daily concentration in a city was the arithmetic mean of the data collected by the two different pollen traps located in the area of the city.

### Pollutants

We collected further data from ARPAV for the pollution in Verona and Padua. Data were provided by a network of air analyzers homogeneously positioned in both cities. Information for Padua and Verona was obtained by two environment control units for each city. Pollutants specifically monitored were: ozone (acceptable levels ​< ​120 ​μg/mˆ3) and PM10 (acceptable levels: 50 ​μg/mˆ3) NO2, SO2 and CO.^13^

### Statistical analyses

Data were analyzed by using chi-square test with a significance level of 5%. R software was used. Verona and Padua data were first analyzed separately; as no significant differences could be observed concerning the investigated variables within the considered timeframe (data not shown), the two databases have been gathered and re-analyzed as a single population. Data from the 2013 to 2015 timeframe were cumulatively analyzed, as no significant differences between the three analyzed years were detected.

## Results

**Fig. 1:**
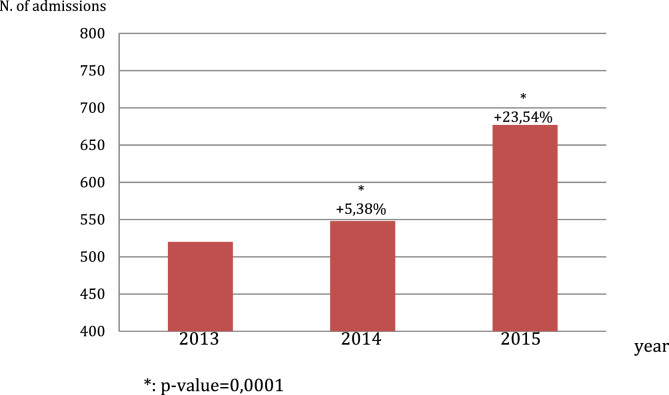
Trend of ER admissions for asthma within the considered timeframe.

Overall, 1745 ​ER admissions for asthma were registered in the considered timeframe. A persistent and significant increase of ER admissions for asthma has been registered from 2013 to 2015, as shown by Fig. 1.

**Fig. 2:**
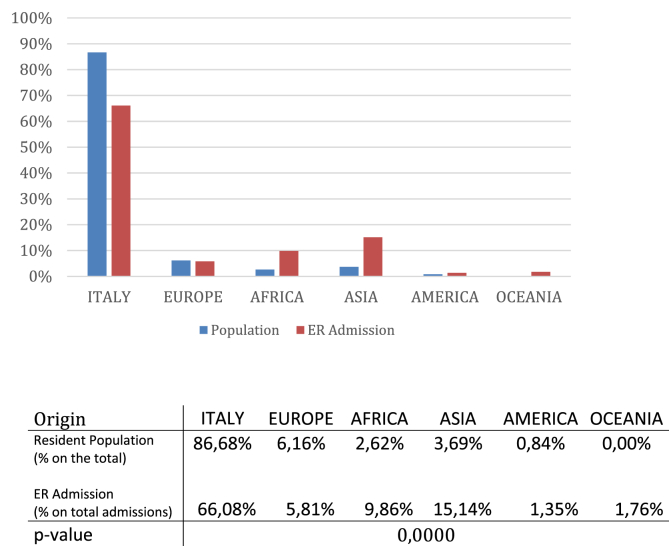
ER admissions vs residents stratified by origin.

A slight but not significant prevalence of females and patients over 50 years of age has been observed (p-value: 0,0514). Immigrants accounted for 32%, 36% and 26% of admissions respectively in 2013, 2014 and 2015. The prevalence of immigrants' admissions was significantly higher when comparing the relative ratio of immigrant populations/Italian nationals living in the towns of Verona and Padua (p ​< ​0.05) (Fig. 2). People coming from Asia an Africa showed the higher number of admission.

A minority of patients (21.20%) had more than one admission to ER during the study period. When comparing the last subgroup of patients with the others, no significant differences can be described in terms of age (p ​= ​0,2904), gender (p ​= ​0,3195), origin (p ​= ​0,5735), and asthma attack severity (p ​= ​0,4137).

**Fig. 3:**
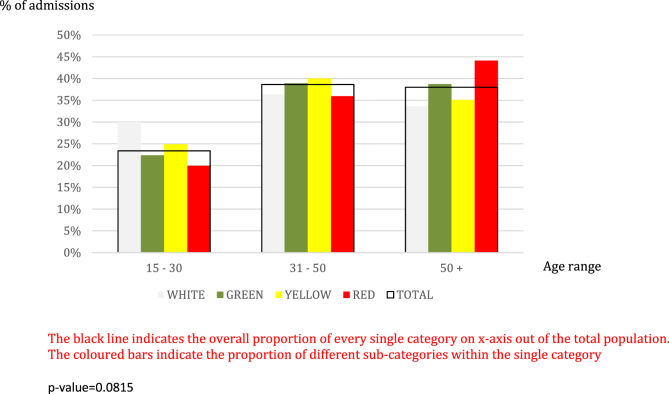
Admission codes clustered by age range.

**Fig. 4:**
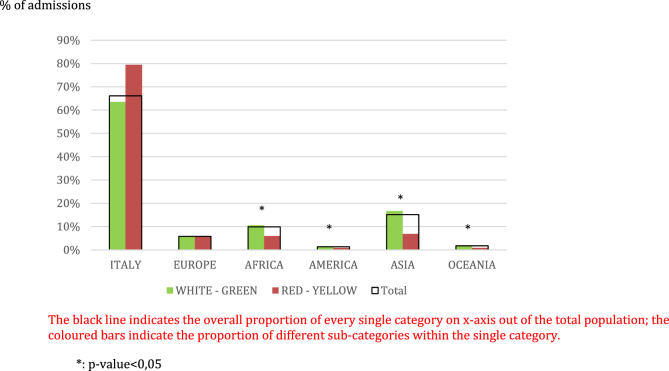
Admission codes clustered by origin.

The admissions were coded as follows: white, 6.30%; green, 35.36%; yellow, 39.37%; red, 18.97%. No significant differences were recorded when stratifying the admission codes by gender (p ​= ​0,6528). People aged ≥50 years were more frequently admitted with a red code, but the trend was not statistically significant (p ​= ​0,0815) (Fig. 3). By contrast, amongst immigrants there was a higher prevalence of white and green codes observed in comparison with Italian nationals (Fig. 4).

**Fig. 5:**
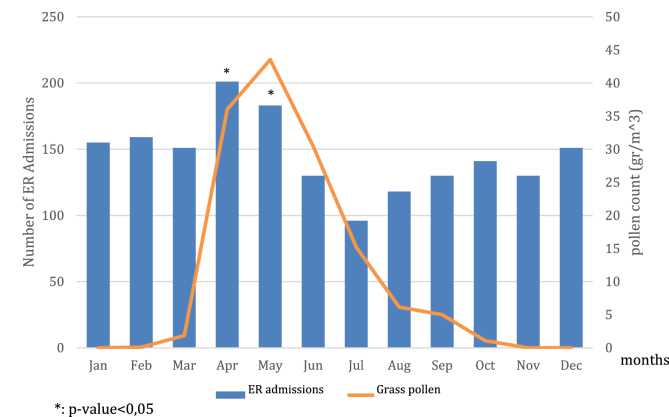
Trend of admissions during the whole year, paired with grass pollen count.

When analyzing the distribution of admissions during the whole year, a peak in April and May was observed, in comparison with the other months (p ​= ​0.0000). In the same period a significant increase of grass pollen count was recorded (Fig. 5). There was no significant correlation between admission clusters and other pollens of mold spore peaks (data not shown). As far as pollutants are concerned (Table 1), during the study timeframe the level of ozone (O_3_) exceeded the acceptable limits for 63 days. The average number of ER admissions was comparable to the days when O_3_ was in the normal range (p ​= ​0,9368). By contrast, a not negligible increase of the average ER admissions was observed at the same time as PM_10_ level was above the reference range (p ​= ​0,0053).

**Table 1:** Average daily ER admissions by PM_10_ and O_3_ levels.

Pollutants (μg/m^3^)	days	Daily ER admissions (mean)	p-value
O_3_≤120	2129	0.8027	0.9368
O_3_ ​> ​120	63	0.7937	
PM_10_≤50	1678	0.7729	0.0053
PM_10_ ​> ​50	514	0.8988	

## Discussion

Our retrospective analysis showed that ER admissions for asthma exacerbations between 2013 and 2015 in Northeastern Italy significantly increased year by year, with a slight but not significant prevalence of females and patients over 50 years of age. Overall, the immigrants' admissions were more frequent, and amongst the non-European residents a higher prevalence of white and green codes was observed. Grass pollen peak and PM_10_ high levels represented environmental determinants of ER admissions increase.

Despite a number of treatment options, asthma control, even in diagnosed and treated patients, still represents an unmet need.^1^, ^8^, ^14^ The first determinant of lack of control is non-adherence to the treatment.^15^, ^16^ The high proportion of white-green codes suggests that ER admissions are related to poorly controlled and not to truly severe asthma, as highlighted by a general practitioner large database analysis reporting an increased risk of asthma related hospital admissions in mild to moderate uncontrolled asthma.^8^ Similarly, fatal asthma cases described by our group were related to uncontrolled disease, misuse of drugs and lack of asthma emergency plan more than to asthma severity.^9^ By contrast, according to a large UK and US study, the ER admission rate and costs in the GINA step 5 group approximately doubled the rate and costs in the GINA step 1 population.^17^ This observation is not reported by other studies which highlighted the poor asthma control due to low treatment adherence, and the inadequate knowledge about self-management of an asthma attack as major determinants of asthma-related emergency department admissions in the adult population, in accordance with our results.^15^, ^16^ Moreover, Steppuhn et al described a higher prevalence of exacerbations requiring emergency room in asthmatic patients with comorbidities including allergic rhinitis, gastroesophageal reflux disease, and acetylsalicylic acid exacerbated respiratory disease (AERD), the latter being particularly relevant.^18^ These findings further support that implementing a regular follow-up schedule and providing an asthma and related comorbidities action plan may allow preventing avoidable ER admissions.

As far as demographic determinants are concerned, the slight although not significant prevalence of females in our dataset is consistent with other reports^19^; the late onset asthma phenotype, more common in women and frequently associated with a more difficult disease control, may account for this observation.^20^ In accordance with our results, a preponderance of elderly was described^19^ as a determinant of re-admission as well.^21^ In our population a higher proportion of red codes was detected among older patients. Concomitant diseases such as hypertension, ischemic heart disease or diabetes may account for a more severe clinical presentation. The relevance of ethnicity in the asthmatic population seeking for emergency room has not been specifically investigated by previous reports. It is known that ethnicity contributes to asthma phenotype definition,^22^ which may be associated with a more unstable disease. Nevertheless, the more difficult access to Health Service facilities, as previously reported,^23^ probably provides the main explanation to the overall higher rate of immigrants' admissions, when comparing the relative ratio of immigrant populations/Italian nationals living in the towns of Verona and Padua, that we observed in our dataset. In fact, amongst the non-European residents a higher prevalence of white and green codes was observed. This finding suggests a potential difficult interaction between immigrants and primary care, including General Practitioners (GPs) and Pharmacies, perhaps due to linguistic and cultural barriers. Furthermore, at least in Italy, the right to a regular General Practitioner implies obtaining a residence permit. It may happen that if delays in that process occur, immigrants may not have a direct referral to a GP, and the ER may become the first line of Health care Service for them.

Several studies reported a significant relationship between the level of pollution and the number of ER admissions due to asthma exacerbations.^24^, ^25^, ^26^, ^27^ Peel et al.^26^ observed a significant association between peaks of several correlated gaseous and particulate pollutants levels, including ozone, nitrogen oxide (NO2), carbon monoxide, particulate matter (PM), and organic carbon, and an increase of ER visits for respiratory diseases exacerbations. Of note the authors considered different respiratory conditions besides asthma, including chronic obstructive pulmonary disease, upper respiratory infection, and pneumonia. When looking at the studies specifically addressing asthma, among the pollutants ozone and PM10 were reported to be more frequently responsible for the increase of asthma exacerbation rate, not only but mostly in the pediatric population.^25^, ^26^ In our study ozone PM_10_, NO2, SO2 and CO were analyzed; PM_10_ represented the only determinants of ER admissions increase among the environmental pollutants. Of note our dataset included adult patients only, and a different age related bronchial hypersensitivity to pollutants might be hypothesized. Furthermore, looking at the published literature on the topic, it is not surprising that the clinical relevance of different pollutants varies in relation to the specific regional ambient. Erbas et al.^25^ demonstrated that within the same city PM10 or NO2 and ozone revealed the strongest association with increased childhood asthma ER presentations in the Central and Western districts respectively.

According to our findings, a significant increase of ER admissions for asthma paralleled the grass pollen count peaks during the springtime. Although a seasonal distribution of ER admissions had been already reported,^19^ molds and especially Alternaria among the inhalant allergens were described in the literature as major determinants of asthma exacerbation.^28^, ^29^, ^30^ By contrast, in our study no admission clusters were detected during the Alternaria season (July–September), despite a high presence of mold spores in our region during the same period.^12^ The different populations included in the published studies may provide a possible explanation for that. In fact, most of the reports highlighting Alternaria as a trigger of summer and autumnal asthma exacerbations requiring ER admission included subjects aged below 18 years,^28^, ^29^, ^30^ differently from our study, and an age-dependent sensitivity to environmental factors has been described.^19^, ^27^ Also, the complex interactions between inhalant allergens, meteorological factors, including wind and thunderstorm, and pollutants levels in a specific geographic districts, may modify the final effect of the same trigger.^25^, ^31^ Furthermore, data on sensitization to inhalant allergens are lacking in our dataset, so that the allergic profile of our study population, potentially different from others, cannot be evaluated as a determinant.

Some limitations of our study have to be highlighted. The retrospective design may hamper the strength of results. Furthermore, some patient-related information such as atopic status, the presence of comorbidities and details concerning the specific asthma phenotypes are not included in the dataset, due to the difficult collection in the ER daily routine setting, in the absence of a prospective study design. For the same reasons, the diagnosis of asthma was mainly based on the clinical history and presentation, without any further specific assessment. Thus, the possibility of misdiagnosis, particularly in the elderly, cannot be completely ruled out.

To the best of our knowledge, however, this is the first study specifically investigating patient-related and environmental-related determinants of ER admissions for asthma in Italy, and one of the few published reports on the topic. As previously discussed, the relevance of potential environmental triggers of asthma exacerbations cannot be generalized and should be investigated within a specific geographical district, in which the complex interaction of climatic variables,^25^, ^31^ as well as the different populations' characteristics^19^, ^27^ may modulate the clinical impact of the same determinant.

Although not free from potential bias, our observation may provide practical help in identifying asthmatic patients with a higher risk for asthma related ER admission and in orienting preventive strategies accordingly. The systematic referral to a specialized center after the discharge from the ER, although not so easy to realize, may represent a way for implementing acute asthma management, prevent further attacks and avoid ER readmission. Due to the relevance of the local environmental setting and it impact on asthma control, further similar research should be conducted across different countries in order to identify specific determinants.

## Conclusions

Despite a number of treatment alternatives, asthma control still represents an unmet need even in diagnosed and treated patients. Investigating the traits of patients referring to the ER for asthma exacerbations may help in identifying at-risk individuals. It has been demonstrated that a regular follow-up visit schedule is able to reduce the risk for asthma exacerbation requiring admission.^32^ Thus, implementing patient education in order to improve treatment adherence, as well as providing an asthma action plan to every asthmatic patient, continue to be the most urgent needs.

## Declarations

### Ethics approval and consent to participate

Ethics approval was obtained from the Medical Direction and Regional Ethics Committee.

### Consent for publication

Not applicable.

### Availability of data and materials

Data sharing is not applicable to this article as no datasets were generated or analysed during the current study.

### Funding

Not applicable.

### Conflicts of interest

The authors declare no conflicts of interest.
